# Transcriptomic profile comparison of monocytes from rheumatoid arthritis patients in treatment with methotrexate, anti-TNFa, abatacept or tocilizumab

**DOI:** 10.1371/journal.pone.0282564

**Published:** 2023-03-06

**Authors:** Maria Talmon, Marcella Percio, Joyce Afrakoma Obeng, Federico A. Ruffinatti, Daniele Sola, Pier Paolo Sainaghi, Emanuela Bellis, Stefano Cusinato, Aurora Ianniello, Luigia G. Fresu

**Affiliations:** 1 Department of Health Sciences, School of Medicine, University of Piemonte Orientale, Novara, Italy; 2 Department of Life Sciences and System Biology, University of Torino, Torino, Italy; 3 Struttura Complessa Allergologia ed Immunologia, CAAD Ipazia, Azienda Ospedaliero-Universitaria Maggiore della Carità, Novara, Italy; 4 Department of Translational Medicine, School of Medicine, University of Piemonte Orientale, Novara, Italy; 5 Day Hospital Multidisciplinare—Struttura Complessa di Nefrologia e Dialisi, Ospedale di Borgomanero, Borgomanero, Italy; Medical Center - University of Freiburg, GERMANY

## Abstract

It is well documented that patients affected by rheumatoid arthritis (RA) have distinct susceptibility to the different biologic DMARDs available on the market, probably because of the many facets of the disease. Monocytes are deeply involved in the pathogenesis of RA and we therefore evaluated and compared the transcriptomic profile of monocytes isolated from patients on treatment with methotrexate alone or in combination with tocilizumab, anti-TNFα or abatacept and from healthy donors. Whole-genome transcriptomics yielded a list of regulated genes by Rank Product statistics and DAVID was then used for functional annotation enrichment analysis. Last, data were validated by qRT-PCR. Abatacept, tocilizumab and anti-TNFa cohorts were separately compared with methotrexate, leading to the identification of 78, 6, and 436 differentially expressed genes, respectively. The upper-most ranked genes were related to inflammatory processes and immune responses. Such an approach draws the genomic profile of monocytes in treated RA patients and lays the basis for finding gene signature for tailored therapeutic choices.

## Introduction

Rheumatoid arthritis (RA) is a systemic inflammatory and autoimmune disorder of unknown aetiology, characterized by chronic inflammation of the synovium and erosions preferentially involving peripheral joints [1, 2]. If untreated, RA significantly reduces the quality of life of patients and leads to an increase in mortality [3, 4]. Current pharmacological approaches include nonsteroidal anti-inflammatory drugs (NSAIDs), which just mitigate pain and stiffness, corticosteroids and synthetic Disease-Modifying AntiRheumatic Drug (sDMARD), including methotrexate (MTX), which have anti-inflammatory and immunosuppressive effects [5], and numerous biological DMARDs (bDMARDs). The first biologics targeted solely TNFα, but other agents with different mechanisms of action are now available [6, 7] including targeting cytotoxic T-lymphocyte-associated protein 4 (abatacept) and cytokine signalling pathways (e.g. IL-1 and IL-6). bDMARDs control signs and symptoms of inflammation and retard progression of joint destruction by similar efficacies despite their different mechanisms of action and it is surprising that there are still no true predictors response to a particular bDMARD.

Monocytes/macrophages are deeply involved in the pathogenesis of RA [8]. According to the expression of CD14 (the LPS co-receptor) and CD16 (the low-affinity receptor for the Fc region of IgG), they can be divided into “classical” CD14++CD16- cells, “non-classical” CD14+CD16+ cells and “intermediate” CD14++CD16+ monocytes [9, 10]. These three monocyte populations have different functional role and the CD14++ subsets are differently present in various inflammatory diseases [11–13]. Importantly, these populations have been shown to change according to disease severity and duration [14, 15]. In particular, intermediate monocytes CD14++CD16+ are highly present in patients with active rheumatoid arthritis and strictly correlate to disease activity [16, 17]. Furthermore, we have previously demonstrated a positive correlation between the percentage of CD14+ CD16+ circulating monocytes and DAS28 and US7 scores [18]. Thereafter, the phenotype of monocytes/macrophages have been postulated to correlate with the responsiveness to pharmacological treatment with different synthetic or biological DMARDs (bDMARD) [19–21]. We have previously observed [22] that etanercept, tocilizumab and abatacept differ in their ability to modulate human monocytes and monocyte-derived macrophages from healthy volunteers in vitro, in terms of reactivity and phenotype. In particular, tocilizumab appears to be more effective in inducing an anti-inflammatory phenotype of monocytes / macrophages compared to etanercept and abatacept, probably also owing to the increased expression of PPARγ whose anti-inflammatory effects are well recognized [23].

In the present contribution we evaluated the transcriptomic variations in the monocytes of patients affected by RA treated with methotrexate alone or in combination with anti-TNFα, tocilizumab or abatacept.

## Materials and methods

### Patients and healthy subjects

RA outpatients under treatment with methotrexate (MTX, n = 15) alone or in co-treatment with anti-TNFα drugs (anti-TNFα, n = 15), abatacept (ABA, n = 15) and tocilizumab (TOCI, n = 15) attending the immuno-rheumatology clinic and 30 healthy donors (HD, who did not have any arthritis symptoms), were enrolled in this observational pilot study. All subjects gave their informed consent for inclusion before they participated in the study. The study was conducted in accordance with the Declaration of Helsinki, and the protocol was approved by the Ethics Committee of Azienda Ospedaliera Maggiore della Carità, Novara (Prot. 241/CE). The inclusion criteria were: age ≥18 years, established RA (disease duration: 3 years and more) diagnosed according to current ACR/EULAR classification according to the 1987 criteria of the American College of Rheumatology [24], no smoking habit, no other inflammatory and chronic diseases (including hypertension, diabetes, cardio-vascular diseases, hypercholesterolemia, malignancy, bacterial or viral infections). For each patient, a complete medical history was obtained and a full physical examination, including joint assessment, was performed. Clinical assessment routinely included the 28-joint Disease Activity Score, DAS28, erythrocyte sedimentation rate (ESR) and C-reactive protein (CRP) level, Rheumatoid Factor (RF) and anti-citrullinated protein antibodies (ACPA) [25]. Blood samples were obtained from each participant at fasting at 9.00 a.m.

### Monocyte isolation

Monocytes were isolated from healthy donors and RA patients by standard technique of dextran sedimentation and Histopaque (density = 1.077 g cm^−3^, Sigma-Aldrich Milan, Italy) gradient centrifugation (400×g, 30 min, room temperature) and recovered by thin suction at the interface, as described previously [26]. Purified monocytes populations were obtained by adhesion (90 min, 37°C, 5% CO_2_) in serum free RPMI 1640 medium (Sigma-Aldrich, Milan, Italy) supplemented with 2 mM glutamine and 1% penicillin-streptomycin. Then, non-adherent cells (mainly lymphocytes) were removed by gentle washing. Cell viability (trypan blue dye exclusion) was usually >98%.

### Superoxide anion (O_2_^−^) production

Superoxide anion production was evaluated by the superoxide dismutase (SOD)-sensitive cytochrome C reduction assay. Monocytes (1×10^6^ cells/plate) were incubated with 1 mg/ml Cytocrhome C (Sigma-aldrich) for 30 min 37°C and then the substrate reduction was evaluated at the spectrophotometer (550 nm). Results were expressed as nmoles cytochrome C reduced/10^6^cells/30 min, using an extinction coefficient of 21.1 mM.

### Flow cytometry analysis

Expression of surface markers CD14 and CD16 on monocytes from RA patients and healthy volunteers was evaluated on the day of collection, by flow cytometry (Attune NxT Flow Cytometer–Thermo Fisher Scientific, Monza, Italy) and analysed by FlowJo™ Software (BD Bioscience, Oxford, UK). Cells were incubated (1 h, at 4°C, in the dark) with saturating concentrations of allophycocianin (APC)-conjugated human anti-CD14 monoclonal antibody (Thermo Fisher, Monza, Italy) and fluorescein isothiocyanate (FITC)-conjugated anti-CD16 monoclonal antibody (Thermo Fisher, Monza, Italy) and then washed twice in phosphate-buffered saline (PBS). Monocytes were analysed by selective gating based on forward and side light scatter, as described [27].

### RNA isolation and whole-genome microarray

Gene expression profiling was performed on primary monocyte cultures from a total of 31 samples, according to the following experimental design: 10 samples from healthy patients, 6 samples from MTX-, 5 samples from abatacept-, 5 samples from anti-TNFα- and 5 samples from tocilizumab-treated patients. For each sample, total RNA was isolated by Tri-Reagent (Life Technologies, Monza, Italy) from 5×106 freshly isolated monocytes. The amount and purity of total RNA was quantified at the spectrophotometer (Nanodrop, Thermo Fisher, Monza, Italy) by measuring the optical density at 260 and 280 nm.

The samples were subject to one-color gene expression profiling with Agilent SurePrint G3 Human Gene Expression (GE) v3 8x60K Microarray (Agilent Technologies, Santa Clara, CA, USA). Each chip featured a total of 58,201 distinct biological probes. Slides were scanned with an Agilent C dual-laser microarray scanner and images analysed with Agilent Feature Extraction software. Raw data were background subtracted using *normexp* algorithm and then normalized through the *quantile-quantile* between-array normalization procedure. Prior to statistical analysis, probes corresponding to long non-coding RNA (lncRNA), as well as unannotated genes (no Gene Symbol or Gene Name) were excluded to focus on annotated coding transcripts. In addition, only those probes exhibiting an absolute expression (log_2_ intensity value) greater than 6 in 80% (or more) of the samples, in at least one group, were retained for subsequent analysis. Overall, such a filtering procedure returned a final dataset of 25,721 distinct probes. Finally, a two-class rank product statistic [28–30] was used for significance assessment, through RankProd/Bioconductor package (ver. 3.8.0) [31, 32]. *P*-values were adjusted for multiple testing by using Benjamini-Hochberg (BH) False Discovery Rate (FDR) correction [33] and every gene with an adjusted *p*-value smaller than 0.05 was considered statistically significant. Upregulated genes featuring a Fold Change (FC) less than 1.5 and downregulated genes featuring a FC greater than 1/1.5 ≈ 0.6667 were rejected a posteriori and expunged from Differentially Expressed Gene (DEG) lists. Functional annotation was performed using DAVID Bioinformatics Resources ver. 6.8 (https://david.ncifcrf.gov/) [34]. BH-FDR correction was applied to enriched GO terms and KEGG pathways, again using an adjusted *p*-value cut off equal to 0.05.

The data discussed in this publication have been deposited in NCBI’s Gene Expression Omnibus and are accessible through GEO Series accession number GSE224330 (https://www.ncbi.nlm.nih.gov/geo/query/acc.cgi?acc=GSE224330).

### Quantitative real-time PCR (qRT-PCR)

Validation of the microarray results by qRT- PCR was performed on 10–15 samples for each group of donors. RNA was isolated by Tri-Reagent (Life Technologies, Monza, Italy). cDNA synthesis was performed using a high-capacity cDNA reverse transcription kit (Applied Biosystems, Monza, Italy) according to the manufacturer’s instructions. A two-step cycling real-time PCR was carried out in a volume of 10 μl per well in a 96-well optical reaction plate (Biorad, Milan, Italy) containing SensiFast No-ROX kit (Bioline, London, UK) 1x, forward and reverse primer 400 nM, and 1 μl of cDNA template. The primers used were reported in S1 Table. Results were reported as log_2_FC.

### Data and statistical analysis

Statistical analysis was performed using GraphPad Prism 6. Data are expressed as mean ± SEM of n independent experiments performed in triplicate. All samples were first tested for normality (Shapiro–Wilk test) and homoscedasticity (Levene’s tests). Whenever possible, statistical significance was assessed by parametric tests (i.e., *t*-test or one-way ANOVA, followed by Tukey’s HSD post hoc test). Otherwise, non-parametric alternatives were used, as detailed in the figure legends. Statistical significance was defined as *p*-value < 0.05.

## Results and discussion

### Clinical characteristics of the RA patients

Baseline characteristics of the enrolled healthy donors and RA patients are shown in Table 1.

**Table 1:** Characteristics of healthy donors and RA patients.

	HD (n = 30)	MTX (n = 15)	Abatacept (n = 15)	Anti-TNFα (n = 15)*	Tocilizumab (n = 15)
**Age, mean (S.D.), years **	56.7 (5.2)	73 (9.3)	65.8 (8.8)	57.7 (10.2)	59.8 (12.4)
**Female sex, % **	53.3	83.3	73.3	91.3	77.7
**Disease duration, mean (S.D.), years **	-	8 (6.4)	14 (5)	11.2 (7)	21.5 (14.8)
**RF positivity, %**	-	66.7	64.2	41.6	55.5
**ACPA, %**	-	66.7	71.4	41.6	55.5
**DAS-28 score, mean (S.D.) **	-	2.4 (0.7)	2.8 (1)	2.6 (0.3)	2 (0.9)
**VES, mean (S.D.) **	-	19.1 (20.3)	22.1 (14.5)	15.2 (16.2)	12 (12.5)
**PCR, mean (S.D.) **	-	0.34 (0.34)	0.8 (0.6)	0.4 (0.2)	0.6 (0.8)
**Cortisone treatment, (2-5mg/day) %**	-	50	33.3	0	75
**MTX treatment, %**	-	100	100	100	100
**Dosage, mean (S.D.), mg/week**	-	9.4 (1.3)	10.3 (2.9)	10 (2.2)	11.3 (3.2)
**Erosive RA, %**	-	0	89.1	55.5	100
**Comorbidity, %**	-	75%	55.5	33.3	25
**Recurring comorbidities**	-	osteoporosis, arthrosis	osteoporosis, hypothyroidism	osteoporosis	osteoporosis

*etanercept (n = 10), infliximab (n = 3), certolizumab (n = 1), adalimumab (n = 1)

RF: rheumatoid factor; ACPA: anti-citrullinated protein antibody; ESR: erythrocyte sedimentation rate; CRP: C-reactive protein; MTX: methotrexate. RA patients were treated with the indicated bDMARDs plus methotrexate and in some cases cortisone as reported.

All patients on biological therapy were on background methotrexate (MTX) treatment without significant differences regarding the dose. Patients in all groups received low-dose prednisone (2–5 mg/day) except patients on anti-TNFa treatment because they did not require cortisone support at the time of the study. The mean DAS28 score of patients was very similar between all groups. The mean CRP level was higher in patients treated with abatacept while the majority of patients with RF-positive belonged to methotrexate and abatacept group, 66.7% and 64.2% respectively. None of patients on MTX treatment presented bone erosion unlike 100% of the tocilizumab cohort.

### Phenotype and responsiveness of monocytes

**Fig 1:**
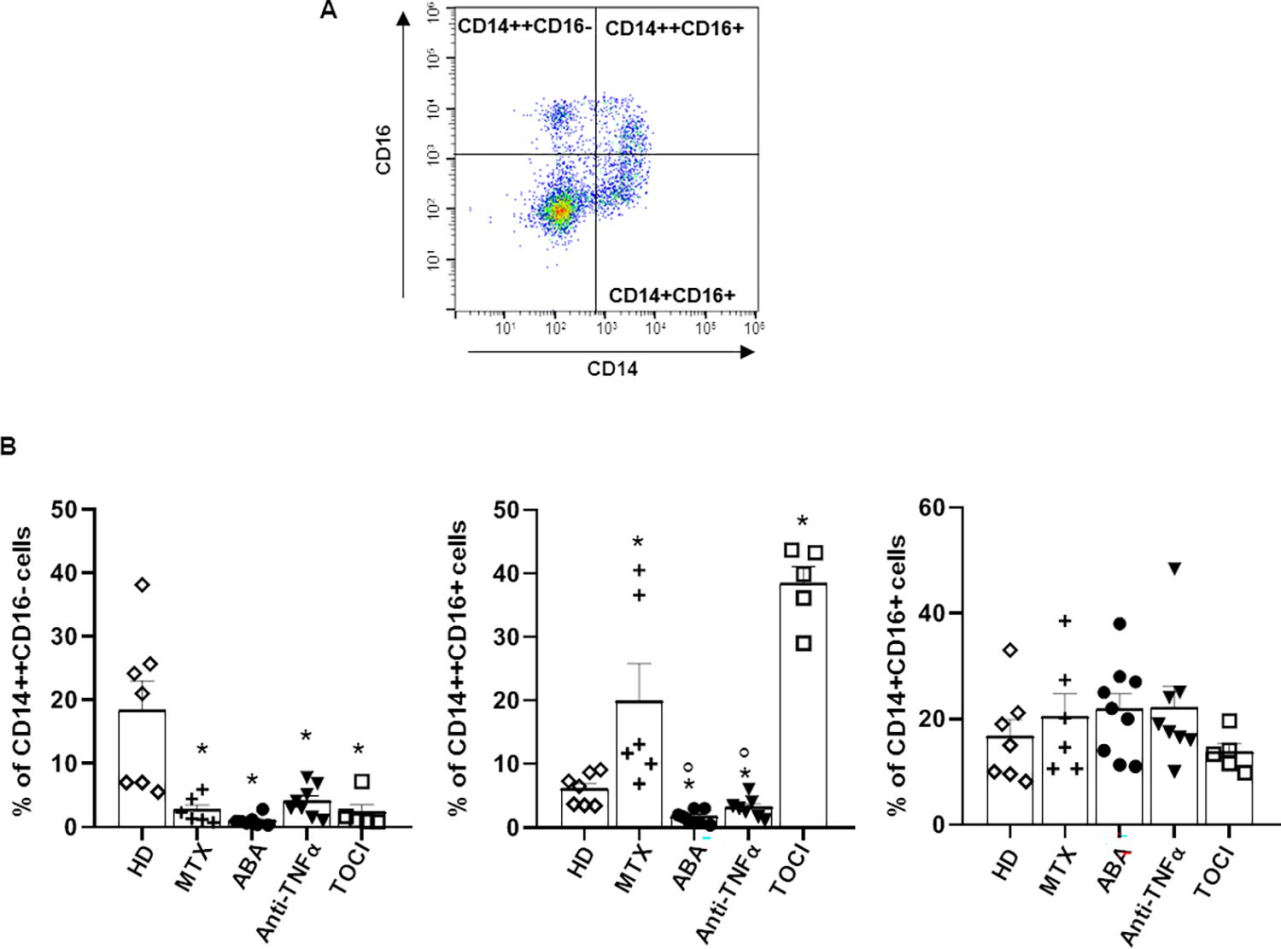
Flow cytometry evaluation of monocytes population from healthy donors and RA patients. A) Representative dot plot of gating strategy. B) Percentage of the indicated populations over CD14/CD16 positive cells. Data are means ± SEM of at least 5 independent experiments (HD = 7; MTX = 8; ABA = 7; anti-TNFα = 9; TOCI = 5) from distinct donors, analysed by one-way ANOVA with Dunnett’s test for multiple comparison. *p-value < 0.05 versus healthy donors (HD); °*p*-value < 0.05 versus MTX (methotrexate). ABA, abatacept; TOCI, tocilizumab.

Freshly isolated monocytes were evaluated for phenotype and basal oxidative stress. Frequency of CD14+/CD16+ monocytes in peripheral blood samples from RA patients and healthy donors was determined by flow cytometry. Fig 1A shows a representative staining pattern of surface expression of CD14 and CD16 on monocytes from a healthy donor in which the three monocyte populations are well distinguished in classical (CD14++CD16-), intermediate (CD14++CD16+) and non-classical (CD14+CD16+).

Fig 1B shows a comparison of the three monocyte populations between RA patients treated with methotrexate, abatacept, anti-TNFa or tocilizumab and healthy donors. The percentage of classical and intermediate monocytes was statistically modulated by the treatments. In fact, all treatments significantly reduced in the same way the percentage of CD14++CD16- compared to healthy donors, while the effects on the intermediate subset CD14++CD16+ was different between drugs: it was high in the methotrexate and tocilizumab patients while it was significantly lower in the abatacept and anti-TNFa patients. The CD14+CD16+ monocyte population presents the same percentage between healthy donors and RA patients under any treatment (Fig 1B).

The distribution of monocytes in the three subsets in RA patients is highly variable between different reports [35], but our results are in agreement with previous data published by others [13, 19, 36, 37], that demonstrated that in RA classical monocytes are decreased compared to healthy donors, and that treatment with both MTX and bDMARDS do not increase this population. Classical monocytes in RA skewed towards intermediate monocytes which are the most representative in RA [13, 14, 17], they are primed to produce several pro-inflammatory cytokines and due to their ability to infiltrate inflamed tissues, can migrate to the synovial lining and differentiate into inflammatory M1 macrophages [35]. Our experiments showed that both abatacept and anti-TNFα are able to significantly decrease them demonstrating an important immunomodulation while the still higher percentage of them in MTX alone treatment and in TOCI patients may suggest a lower anti-inflammatory effect of these drugs on monocyte phenotype, as previously demonstrated [19, 38–40].

It should be underlined that the patients of all groups, except anti-TNFa, are in treatment with glucocorticoids which could influence our results. The ability of glucocorticoids to reduce CD16 expression is known [41, 42] but at higher doses than those used in the patients enrolled in our study. Therefore, the differences in the percentage of CD14/CD16 populations are due to the single drug representative of the group.

**Fig 2:**
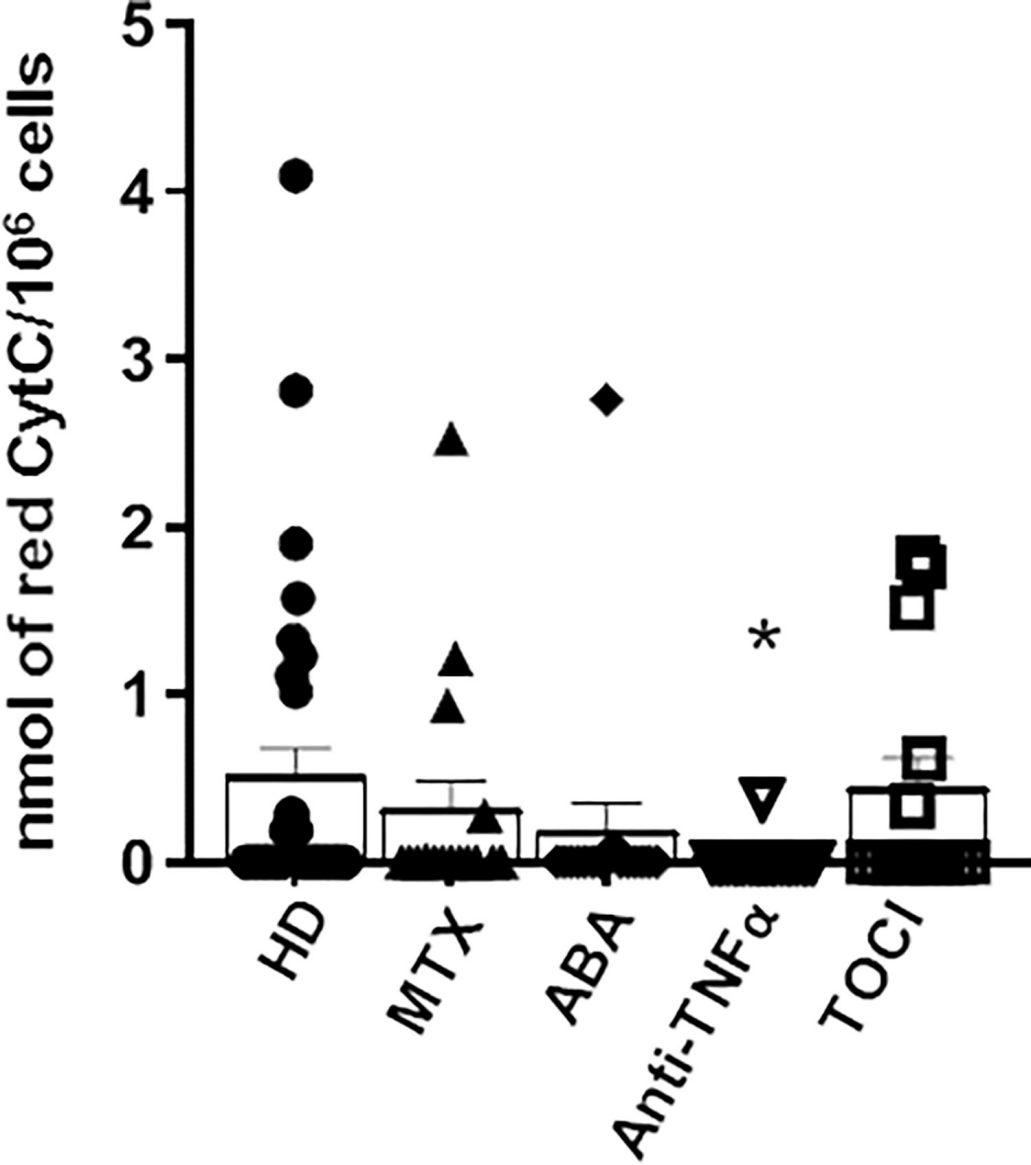
Basal oxidative stress in monocytes from healthy donors and RA patients. Freshly isolated monocytes were analysed for basal production of O_2_^-^. Data are means ± SEM of at least 15 independent experiments from distinct donors (n = 30 healthy control, n = 15 each group of RA patients), analysed by analysed by one-way ANOVA with Dunnett’s test for multiple comparisons. *p-value < 0.05 versus healthy donors (HD). MTX, methotrexate; ABA, abatacept; TOCI, tocilizumab.

Respiratory burst is the rapid response of monocytes to an insult [43] and is therefore representative of their activated state. Then, to ascertain the functionality and responsiveness of the cells under study we evaluated the basal oxidative stress produced by monocytes from healthy donors and patients. Only monocytes from patients treated with TNFα inhibitors showed a significantly reduced production of superoxide anion (Fig 2), in line with the phenotypic analysis.

These results confirm our previous work [22] in which abatacept and etanercept in vitro were more efficacious at inhibiting PMA-induced bursts compared to tocilizumab. Moreover, the data are in line with Boyer JF et al. [44] who demonstrated antioxidant response induced by certolizumab pegol in monocytes, that may contribute to the therapeutic effects of these agents in inflammatory disorders.

### DMARD-specific differential gene expression

**Fig 3:**
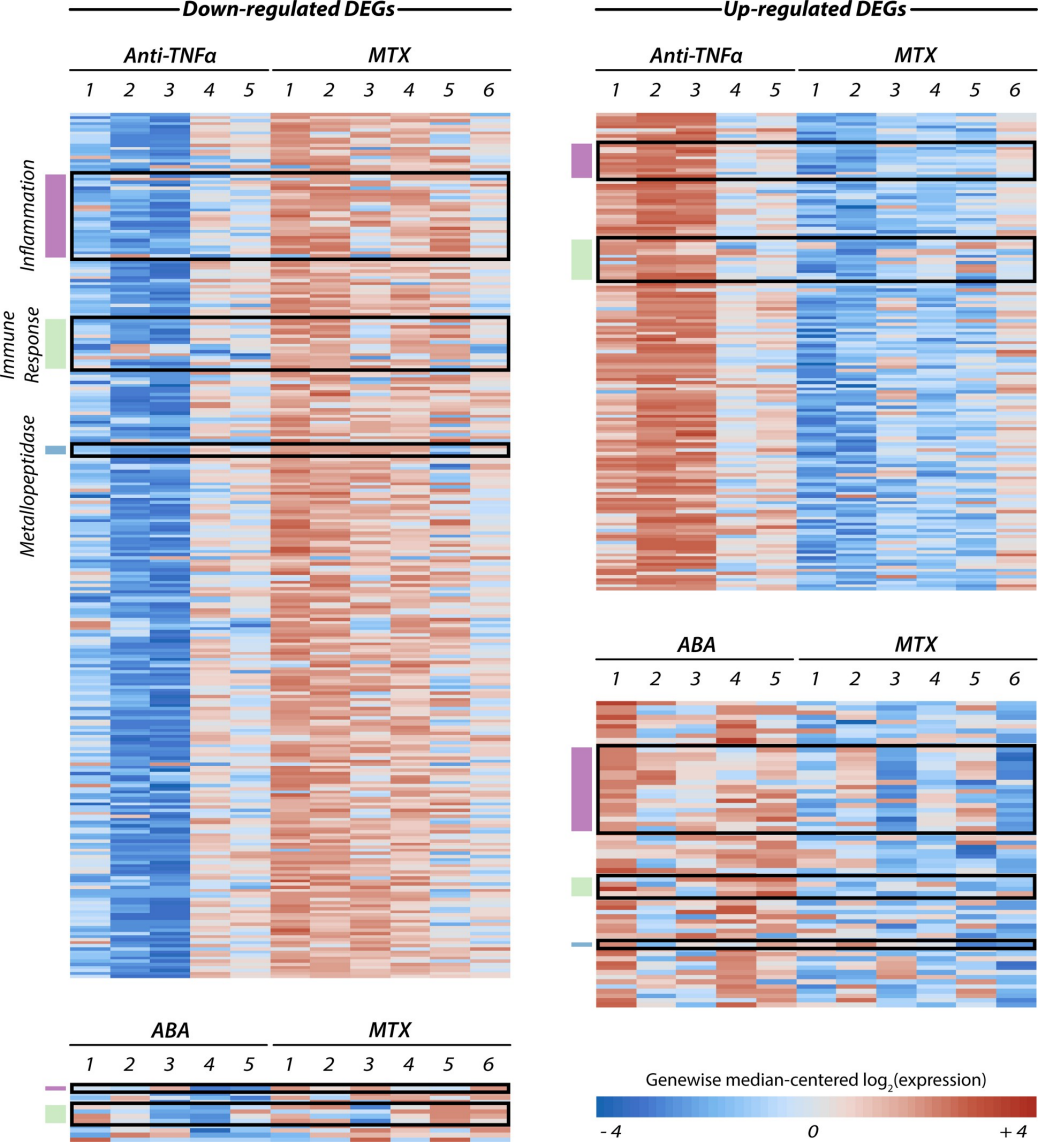
Heat maps showing the transcript levels of the genes significantly modified in mono-cytes isolated from abatacept- (ABA), anti-TNFα-, and MTX-treated RA patients. Each row represents the (log_2_) expression profile of a different DEG centred around its median value, while columns are individual patients. Some relevant functional gene sets are annotated to the left of each map using the following colour scheme: purple for inflammation, green for immune response, and blue for metallopeptidases. The complete list of genes in these categories can be found in S1 File, where the same colour conventions are used.

To further differentiate the actions of the three biologic drugs, we next evaluated the transcriptome of isolated monocytes using Agilent SurePrint G3 Human Gene Expression (GE v3 8x60K) microarrays. Low expression genes were filtered out (see filter details in Materials and Methods section) and all the probes corresponding to unannotated genes or non protein-coding RNAs (such as lincRNAs probes) were discarded, leading to a final set of 25,721 genes. As a first step, the three biologic DMARDs considered in this study were compared to patients treated with methotrexate alone. This led to the identification of 78, 6, and 436 differentially expressed genes (DEGs), respectively (Fig 3).

More in detail, 66 out of 78 DEGs were found to be up regulated in monocytes from patients treated with abatacept compared to the methotrexate group. On the contrary, most of the genes that significantly changed after anti-TNFa therapy appeared to be downregulated, consisting in 155 upward and 281 downward DEGs. As for tocilizumab, our microarray analysis was only able to find 6 (3 up- and 3 down-regulated) DEGs (S1 Fig). In principle, the small number of significant genes resulting from this last comparison could be ascribed either to a similarity between MTX and TOCI in terms of their ultimate molecular targets in monocytes, or to a large heterogeneity of the TOCI samples—possibly because of a higher inter-patient variability—leading in turn to higher gene-wise variances and to a lower statistical power. Inner-group gene-wise average variances (σ^2^_HD_: σ^2^_MTX_: σ^2^_ABA_: σ^2^_Anti-TNFα_: σ^2^_TOCI_ = 1: 0.8: 1.1: 0.8: 1.5)and cross-comparison of MTX and TOCI samples to the control group of healthy donors seem to endorse this last hypothesis.

The aim of this explorative study was to elucidate the transcriptomic signature profile of abatacept, tocilizumab and anti-TNFα on the inflammatory processes that activate monocytes in the pathogenesis of RA and thanks to the analysis performed we have distinguished specific changes induced by the single drugs. For each bDMARD examined, the uppermost ranked genes—in terms of both FC s and *p*-values—were related to inflammatory process and to immune system response.

Specifically, cytokines—such as interleukins, members of TNF and TNF-receptor super-families, CC/CXC chemokine ligands and receptors, granulocyte- and granulocyte-macrophage colony stimulating factor (CSF)—as well as matrix metallopeptidases (MMPs) involved in chemokine/cytokine inactivation, could be found in both ABA- and anti-TNFα-related DEG lists. Likewise, genes encoding for components essential to the function of the immune system such as immunoglobulin polypeptides, cluster of differentiation (CD) proteins and the major histocompatibility complex (MHC), were present in all DEG lists (for the complete set of significantly regulated genes see S1 File). Notably, the most up-regulated gene in both the abatacept and the anti-TNFα cohorts was the same, LRRD1 (leucine-rich repeats and death domain containing 1), possibly involved in the regulation of apoptosis and inflammation upon caspases and NF-κB activation and interaction with TNF-receptors. LRRD1, like other death domain-containing proteins, can also be linked to innate immunity, by interacting with Toll-like receptors.

Despite this noticeable overlap in biological targets, the number of monocytic DEGs actually shared between the patients treated with abatacept and anti-TNFa (respect to the methotrexate group) is just 30, of which only 16 changed in the same direction. Interestingly, this list of concordant DEGs featured only one cytokine (namely the chemokine receptor 1, CCR1) and only two genes directly related to (innate) immune response (i.e. lactotransferrin, LTF, and the already mentioned LRRD1). As shown in the heat map, among the genes specifically modulated by anti-TNFα the vast majority were significantly downregulated. As listed in S1 File, most of them code for molecules involved in the activation of monocytes toward the CD16+ phenotype, such as several adhesion molecules including the chemokine C-X-C motif, the major histocompatibility complex class II-DQA, IL-1 and IL-1R, several members of the TNFa receptors superfamily, and STAT4. The transcriptomic results correlate with the phenotype analysis. In fact, most of these genes negatively regulated by anti-TNFa drugs are usually overexpressed by intermediate monocytes which are significantly reduced by the treatment. This effect is easily attributable to the mechanism of action of anti-TNFα as most of these genes code for players involved in the TNFa-dependent pro-inflammatory cascade [35]. It is interesting to note that also abatacept treatment significantly reduced the intermediate population CD14++CD16+ by altering the molecular profile of monocytes in an opposite manner: abatacept mainly upregulates anti-inflammatory or proapoptotic genes while anti-TNFa drugs mainly induce downregulation of genes that encode for pro-inflammatory proteins. In our opinion this must be ascribed to the specific mechanism of action of the two bDMARDs. While anti-TNFα are drugs targeted versus TNF that is a pleiotropic proinflammatory cytokine mainly produced by activated monocytes and macrophages and, to a lesser extent, by T-lymphocytes, abatacept was developed to reduce T cell responses by limiting CD28 signalling, therefore it is endowed with a more selective mechanism of action and limited to a specific population. However, it has been demonstrated to exert effects beyond T cells, such as in monocytes [39]. Tocilizumab-regulated genes, as detected by this microarray experiment, were too few to allow for any meaningful comparison. However, it is worth noting that the only significant DEG common to all three treatments—even if with different change directions—was PDE4DIP (phosphodiesterase 4D interacting protein, also known as myomegalin), a gene involved in microtubule nucleation and extension from the centrosome to the cell periphery, a crucial process for directed cell migration. Altogether, these observations suggest a high degree of specificity in the mechanisms of action of each biologic DMARD and some common features among the three drugs.

### Enrichment analysis

**Fig 4:**
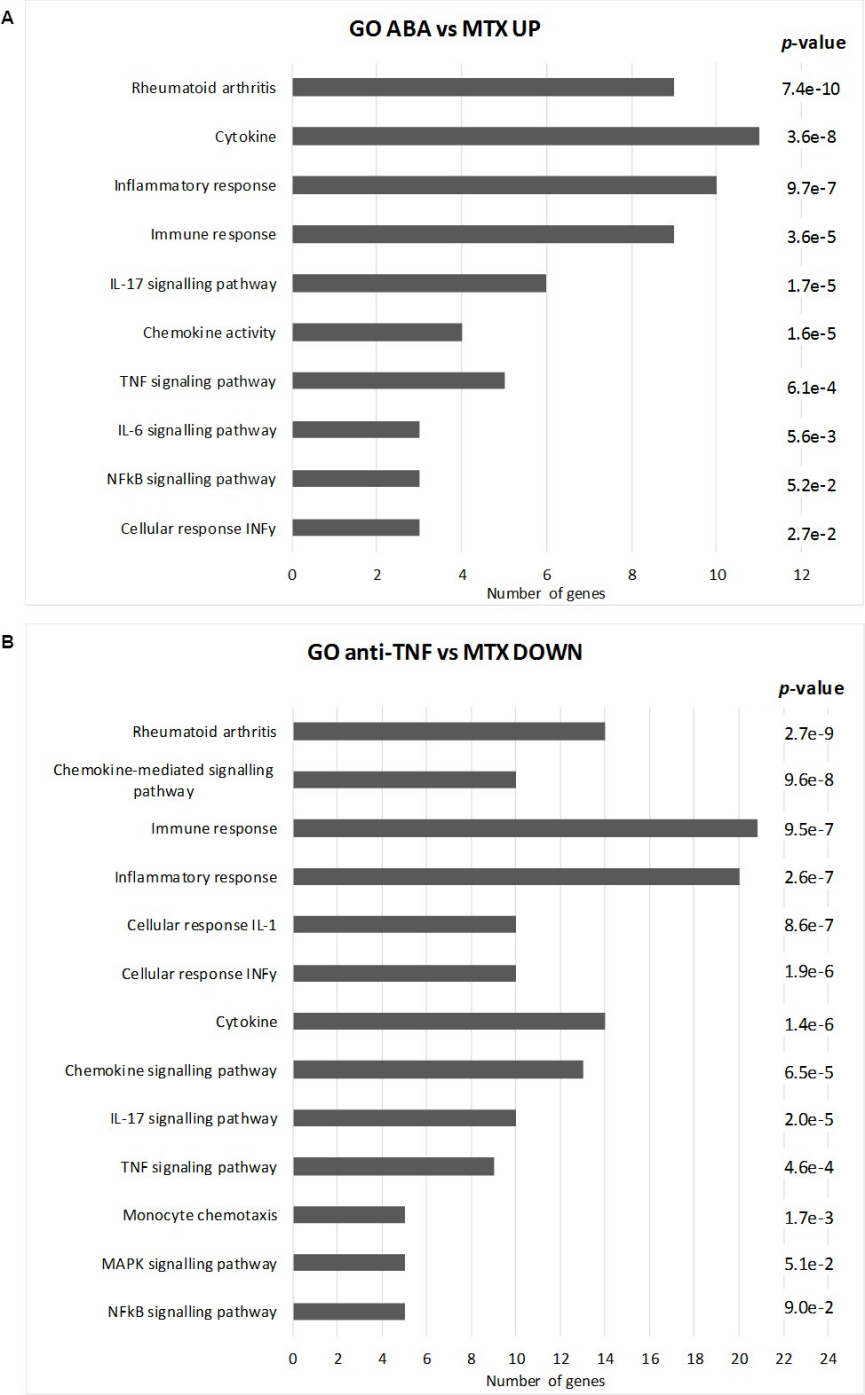
Functional enrichment analysis. Most significant terms, ranked by adjusted *p*-value, as returned by the functional enrichment analysis of the genes up-regulated by abatacept (A, ABA) and down-regulated by anti-TNFα (B) in comparison to methotrexate (MTX). Length of the bars represent the number of genes (x axis) involved in each GO term or pathway.

For an unbiased analysis of the potential pathways and networks involved down-stream of bDMARDs, the DEG lists were uploaded to DAVID Bioinformatics Resources (ver. 6.9) for functional enrichment analysis. Since the algorithm used by DAVID for enrichment analysis requires input lists featuring at least 30 genes in order to get reliable results, only the set of monocytic genes up-regulated in the ABA-group and the lists of up- and down-regulated DEGs in the anti-TNFα group were used, as shown in Fig 4.

Despite the 155 genes found to be significantly up-regulated in monocytes of anti-TNFa -treated patients, no enriched term resulted from functional analysis, that is to say the downregulatory activity of this bDMARD, relative to the methotrexate effect, is somehow noisy (at least in monocytes), not converging to any particular coherent target or cellular pathway. On the contrary, both abatacept up-regulated gene list (66 DEGs) and anti-TNFa down-regulated gene list (281 DEGs) returned a number of meaningful enriched GO-terms (such as *Immune Response*, *Cytokines*, *Inflammatory Response*, *Chemotaxis*, *Cell Migration*) and two common overrepresented signal transduction KEGG pathways: *Rheumatoid arthritis* and *Cytokine-cytokine receptor interaction* (see Fig 4A and 4B for a list of the most relevant enriched terms).

**Fig 5:**
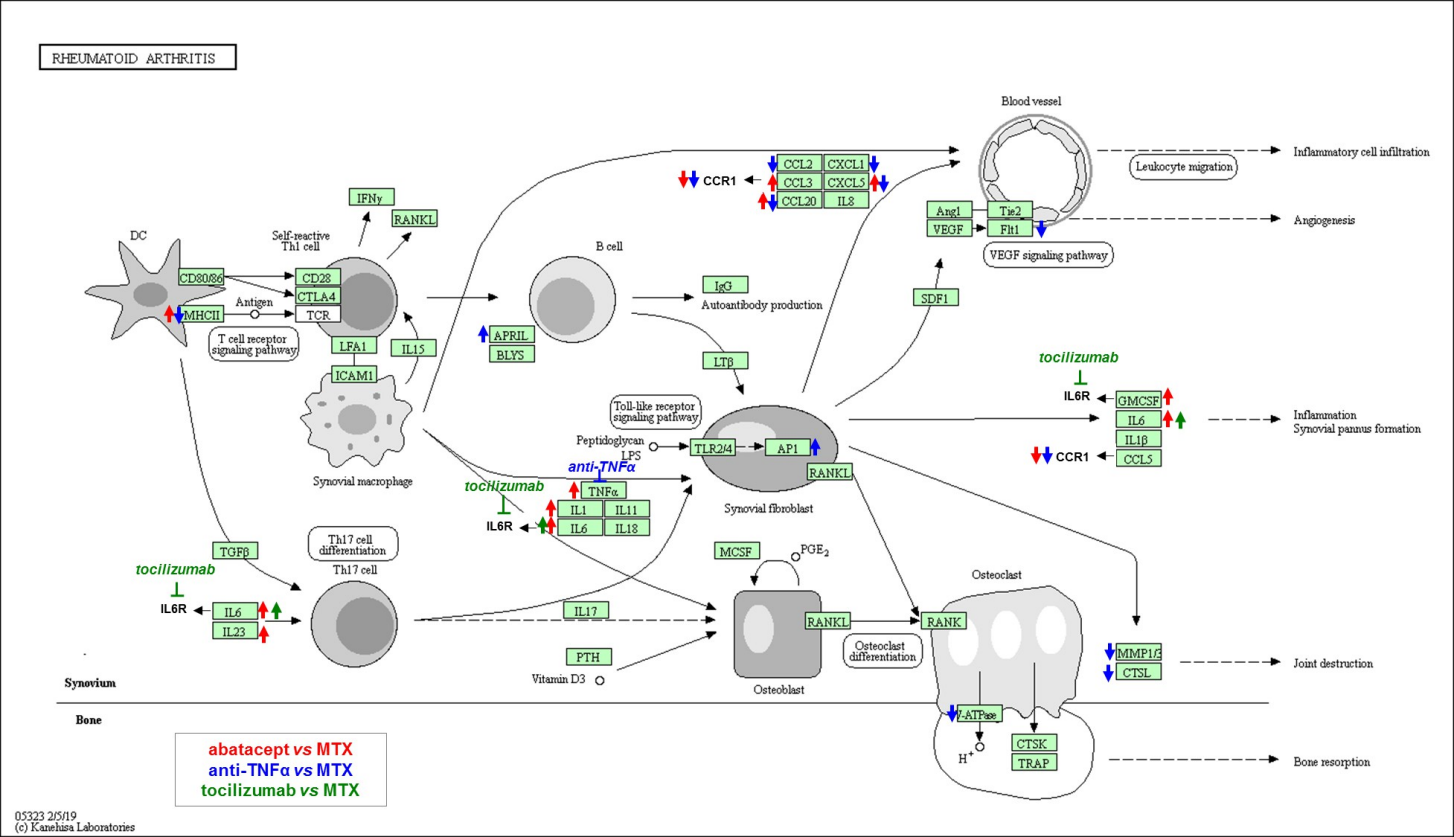
Schematic representation of the transcriptional changes occurred in patients treated with abatacept, tocilizumab and anti-TNFα compared to the methotrexate (MTX). KEGG pathway map hsa05323 *Reumathoid arthritis*-Homo sapiens (human) modified and published under permission of the copyright holder Kanehisa Laboratories.

As a first remark, the biological and clinical relevance of these enriched terms con-firms the reliability of our DEG lists and the effectiveness of the rank product-based statistical procedure. Secondly, as already inferred from the qualitative analysis of the common DEGs (see previous section), functional analysis further showed how the two biologic DMARDs, abatacept and anti-TNFα, worked on similar pathways, but with specific molecular targets and with different (up- or down-regulating) mechanisms of action at the transcriptional level. With particular reference to the KEGG pathway of *Rheumatoid arthritis* [45, 46], Fig 5 shows a schematic map of the transcriptional changes occurring in patients treated with bDMARDs compared to the MTX reference.

### Genes reverted by biologic DMARDs

**Fig 6:**
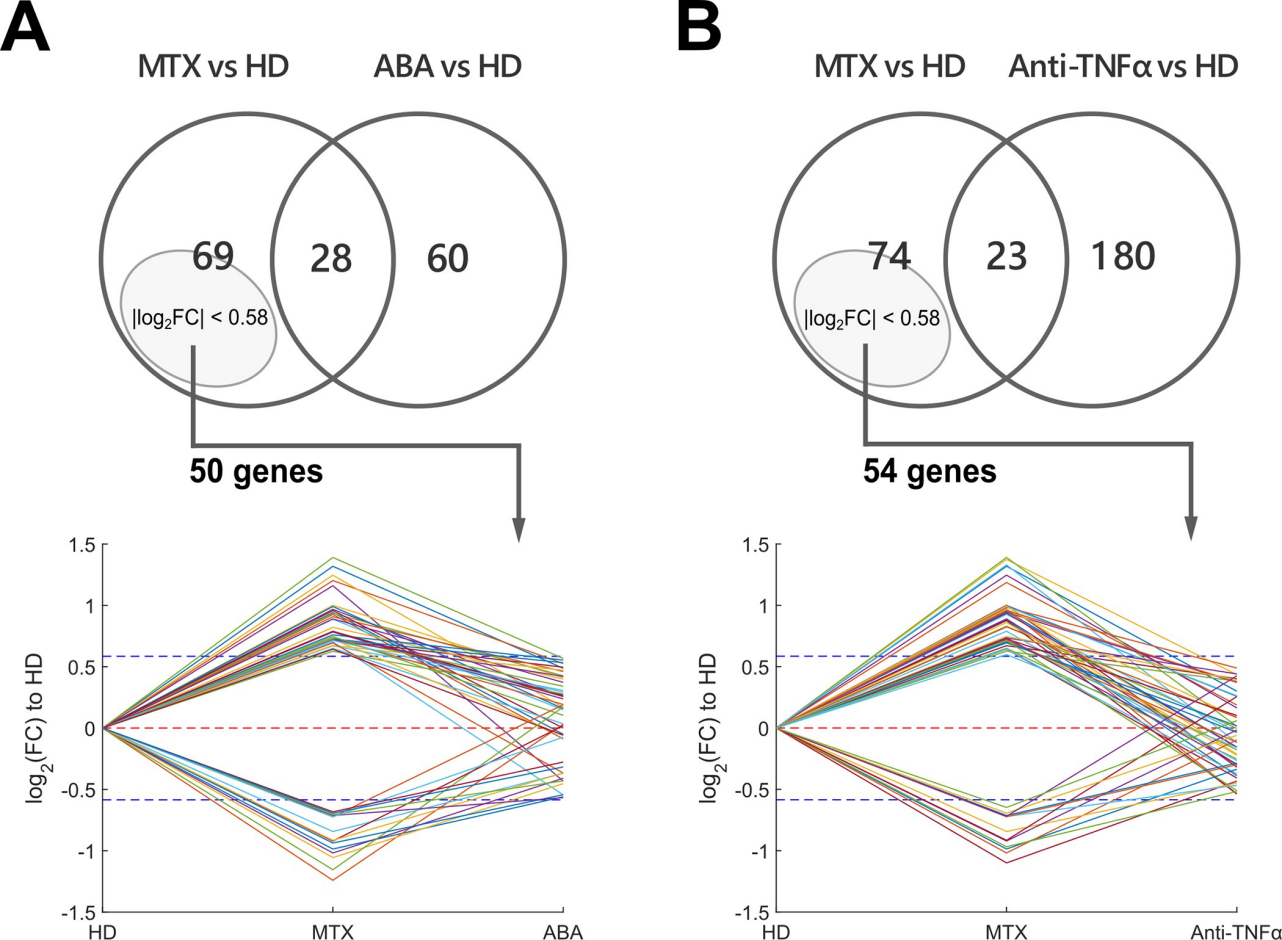
Genes reverted after bDMARD administration. Genes significantly up- and down- regulated by methotrexate (MTX) versus healthy donors (HD) were crossed with genes modulated by abatacept (ABA) and anti-TNFα compared to HD group. (A) For abatacept-treated patients, 69 genes were found to be significantly dysregulated in MTX-treated patients but not after bDMARD administration (left set of the Venn diagram). Among them, only those 50 genes showing a 0.6667 < FC < 1.5 (|log_2_FC| < 0.58) in ABA compared to HD group were deemed to be reverted by abatacept and their log_2_FCs across the different groups are shown using HD as reference. (B) The same procedure applied to detect genes reverted by anti-TNFα.

Using the healthy donor cohort (HD) as a common reference for all the groups of patients, it was possible to extract some information on those genes whose expression in monocytes could be reverted by a particular DMARD. Specifically, genes significantly changed in MTX-vs-HD, but differentially undetectable after biologic drug administration, are likely to be reverted by a particular second-line treatment. However, we are aware that such an analysis may be prone to false detections because of the uncontrolled type II errors (or false negatives), in the bDMARD vs HD comparison and, for this reason, it can only be considered as a preliminary and explorative analysis. Nevertheless, by imposing an additional constraint on FC as a rough measure of the effect size, type II error can be largely mitigated. In particular, only genes detected as DEGs in the sole MTX vs HD comparison and having a 0.6667 < FC < 1.5 in bDMARDs group relative to HD were retained for this analysis. This led to the identification of 50 and 54 genes significantly deregulated in monocytes of methotrexate patients and reverted by abatacept and anti-TNFα respectively (Fig 6).

Just like for the enrichment analysis, even in this case the tocilizumab group was discarded because of its high inter-sample variability leading to few significant DEGs and many false negatives when TOCI group was compared with MTX or HD. Notably, using such a small set of DEGs for reverted-gene detection would have led to erroneously consider a large number of genes as restored by tocilizumab. The complete lists of genes that are likely to have been reverted by abatacept and anti-TNFα can be found in S2 File.

The intersection between ABA and anti-TNF, of the GO terms resulting from the enrichment analysis conducted separately on the 2 sets of reverted genes (S3 File), resulted populated by about 33 genes mostly attributable to the regulation of the immune system and the rheumatoid arthritis pathway (as already discussed for the contrast bDMARD vs MTX). However, we can note how this analysis demonstrates that both bDMARDs, albeit with their already described specificities, work on some common targets related to the immune system and the major histocompatibility complex (e.g, CD79A, CD70, HLA-DQA1, HLA-DQA2, MS4A1, PIKFYVE), but also of other nature, such as for example the SLC32A1 GABA exchanger.

### qRT-PCR validation

**Fig 7:**
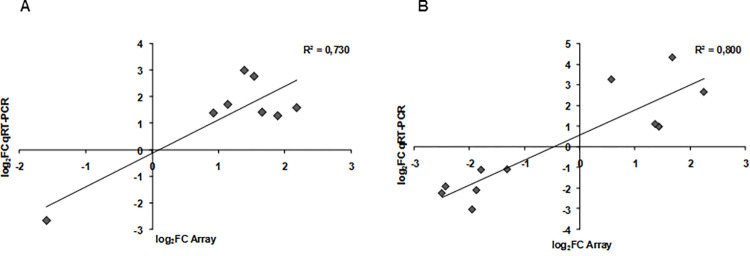
Correlation between average fold change in the expression of selected genes as predicted by microarray analysis and qRT-PCR. Genes up- and down-regulated by abatacept (A) and by anti-TNFα (B). Data are expressed as log_2_FC.

In order to validate microarray results and gain new insight into the transcriptional effects of DMARD, some of the most interesting genes emerged from the previous analysis were selected and validated through real-time qPCR technique. Real-time PCR was performed on 16 selected DEGs modulated by abatacept (IL6, CSF3, NEURL1, TNFα, CSF2, IL1A, PRSS36, CCL4L2, DIRC1, HLA-DQA2, CXCL5, PDE4DIP, KANK1, MMP10, COL9A3 and ADORA2B) and for 8 of these genes (NEURL1, DIRC1, FOS, LEF1, IL6, CCL7, MMP10, ADORA2B, SERPINB2, PPARγ and TNFRSF9) we found a good correlation with the data obtained by microarray (R^2^ = 0.7301; Fig 7A).

Also analysing genes regulated by anti-TNFa we observed a good correlation (R^2^ = 0.8009) between microarray and RT-PCR data. Indeed, 11 genes (NEURL1, DIRC1, FOS, LEF1, IL6, CCL7, MMP10, ADORA2B, SERPINB2, PPARG and TNFRSF9) of the 26 genes analysed (PRSS36, KRTAP19-2, NEURL1, DIRC1, FOS, LEF1, IL6, JUNB, CCL7, MMP10, CXCL5, CCL2, ADORA2B, SERPINB2, KANK1, PPARG, TNFRSF9, COL9A3, CXCL1, MSR1, TNFRSF4, NFKB1, CSF2, IL1RN, PDE4DIP and STAT4) resulted to be significantly modulated at the RT-PCR (Fig 7B). These results demonstrated that the modulation of the expression of genes involved in inflammation and immune response observed by the microarrays analysis were reliable in that they could be replicated by other methods.

### Study limitations

Our findings should be interpreted in light of some limitations of the study. First, the relatively low number of patients enrolled for each arm allowed only to highlight large effect sizes and is prone to type II errors. Nonetheless, the data still represents an important indication of the individual effects of the study drugs on monocyte responsiveness. Second, this is a naturalistic study in which patients were not randomized to the different biologics, and therefore it is likely that selection bias occurred as clinicians chose the different treatments. This naturalistic study also led to some imbalance, including age and gender, in particular in comparison to the healthy donor cohort. Third, the addition of a biologic drug to methotrexate most likely highlights a higher aggressiveness/stage of the disorder, and this disease difference should be taken into account. Yet, we believe that studying the treatment effects over a methotrexate background is a pragmatic approach to the problem in monocytes. While this experimental design has the virtue of representing a realistic clinical scenario (i.e., biologic DMARD administered as a second line treatment after a methotrexate-based standard protocol), results need to be interpreted with caution. Indeed, because of the lack of a real naïve control-group (i.e., RA-untreated patients), any change in gene expression measured after bDMARD administration always relates to the ‘average MTX-induced transcriptional profile’ and different transcriptional effects could be observed if the same bDAMRDs were administered as a first-line therapy.

## Conclusion

This study is an exploratory and hypothesis generating work on the selective responsiveness of monocytes to synthetic and biologic DMARDs. Monocytes are known to be cell mediators and critical components of the inflammation response in rheumatoid arthritis and therefore considered as disease biomarkers [36]. The data show that among bDMARDs, anti-TNFa are those that exert the most prominent activity on monocytes, as reported in Table 2.

**Table 2:** Summary of the effects exerted by methotrexate and bDMARDs on monocyte phenotype and superoxide anion production.

	**Monocyte phenotype**			**Oxidative stress**
** **	**Classical**	**Intermediate**	**Non classical**	
**Methotrexate**	**↓↓**	**↑**	=	=
**Abatacept**	**↓↓**	**↓**	=	=
**anti-TNFα**	**↓↓**	**↓**	=	**↓↓↓**
**Tocilizumab**	**↓↓**	**↑↑**	**↓**	=

Finally, the present contribution demonstrates that it is possible to separate the individual contribution of abatacept and anti-TNFα when administered concomitantly with methotrexate, demonstrating that among the genes deregulated in the methotrexate group, some are specifically restored by abatacept and anti-TNFα.

## Supporting information

S1 Table Sequences of primers used for qRT-PCR. (PDF)

S1 File List of genes significantly regulated in the different contrasts discussed in the paper. Namely: ABA—MTX, anti-TNF—MTX, TOCI—MTX, ABA—HD, anti-TNF—HD, TOCI—HD, MTX—HD.
(XLSX)

S2 File List of genes specifically reverted by ABA and anti-TNF. (XLSX)

S3 File Enrichment of reverted genes. (XLSX)

S1 Fig Heat maps showing the transcript levels of the genes significantly modified in monocytes isolated from tocilizumab- (TOCI) and MTX-treated RA patients. Each row represents the (log2) expression profile of a different DEG centred around its median value, while columns are individual patients.
(TIF)
