# Supplementary material for: Transcriptomic profile comparison of monocytes from rheumatoid arthritis patients in treatment with methotrexate, anti-TNFa, abatacept or tocilizumab
Source: PLoS One. 2023 Mar 6;18(3):e0282564. doi: 10.1371/journal.pone.0282564 (PMC9987802; doi:10.1371/journal.pone.0282564)
Supplement: S1 Table — (PDF) [file pone.0282564.s001.pdf]

Table S1: sequences of primers used for qRT-PCR

Gene		Sequence (5' – 3')
IL-6	Forward	GGAGACTTGCCTGGTGAAAA
	Reverse	GTCAGGGGTGGTTATTGCAT
TNFα	Forward	CATGATCCGGGACGTGGAGC
	Reverse	CTGATTAGAGAGAGGTCCCTG
CCL2	Forward	GAGAGGCTGAGACTAACCCAGA
	Reverse	ATCACAGCTTCTTTGGGACACT
CSF2	Forward	CTCAGAAATGTTTGACCTCCAG
	Reverse	TGACAAGCAGAAAGTCCTTCAG
CSF3	Forward	ATAGCGGCCTTTTCCTCTACC
	Reverse	GCCATTCCCAGTTCTTCCAT
CSF1R	Forward	ATTCATCAACGGCTCTGGCA
	Reverse	AGGACCTCAGGGTATGGGTC
FOS	Forward	TACTACCACTCACCCGCAGA
	Reverse	CGTGGGAATGAAGTTGGCAC
ILR1N	Forward	ATCCAGCAAGATGCAAGCCT
	Reverse	TGACACAGGACAGGCACATC
JUNB	Forward	ACTCATACACAGCTACGGGATACG
	Reverse	GGCTCGGTTTCAGGAGTTTG
LEF1	Forward	CCGAAGAGGAAGGCGATTTAGCT
	Reverse	GCTCCTGAGAGGTTTGTGCTTGTCT
NFKB1	Forward	ACACCGTGTAAACCAAAGCC
	Reverse	CAGCCAGTGTTGTGATTGCT
PPARG	Forward	GCAATCAAAGTGGAGCCTGC
	Reverse	TCTCCGGAAGAAACCCTTGC
STAT4	Forward	GAGACCAGCTCATTGCCTGT
	Reverse	CAATGTGGCAGGTGGAGGAT
TNFRSF4	Forward	CAACTCCTTGATGGTGGCCT
	Reverse	TGGAAGTCATCCAGGGAGGT
TNFRSF9	Forward	AATGGGACGAAGGAGAGGGA
	Reverse	AGAAACGGAGCGTGAGGAAG
TNFRSF15	Forward	AAGGGCCGTCTTCATTTCAGT
	Reverse	TGTGCCCTTGGCTTATCTCC
ADORA2B	Forward	TGTGTCCCGCTCAGGTATA
	Reverse	GGCACTGTCTTTACTGTTCCA
CXCL1	Forward	CAGGGAATTCACCCCAAGAACA
	Reverse	GGATGCAGGATTGAGGCAAGC
CXCL5	Forward	TTCTTCAGGGAGGCTACCAC
	Reverse	AGCTGCGTTGCGTTTGTTTA
DIRC1	Forward	TTGGAGCCTTCCATCATCTC
	Reverse	TCCACCCCCTCAATGAGTTA
	Forward	TACGTGGACCTGGAGAGG

**HLA-DQA1**	**Reverse**	TTGTGTTTTGCCACAGCCA
**IL1A**	**Forward**	ATTGGTCCGATCTTTGACTCT
	**Reverse**	ACTTTGATAACAGTGGTCTCATGG
**MMP1**	**Forward**	TGTCAGGGGAGATCATCG
	**Reverse**	TTCATCAAAATGAGCATCCC
**MMP10**	**Forward**	GGCTCTTTCACTCAGCCAAC
	**Reverse**	GGGAGGTCCGTAGAGAGA
**NEURL1**	**Forward**	CGCATTCTGGGTGGACAA
	**Reverse**	GACCCCGCTGAAGAACAG
**GAPDH**	**Forward**	AACGTGTCAGTGGTGGACCTG
	**Reverse**	AGTGGGTGTCGCTGTTGAAGT
