# Supplementary figures and images for: Transcriptomic profile comparison of monocytes from rheumatoid arthritis patients in treatment with methotrexate, anti-TNFa, abatacept or tocilizumab
Source: PLoS One. 2023 Mar 6;18(3):e0282564. doi: 10.1371/journal.pone.0282564 (PMC9987802; doi:10.1371/journal.pone.0282564)

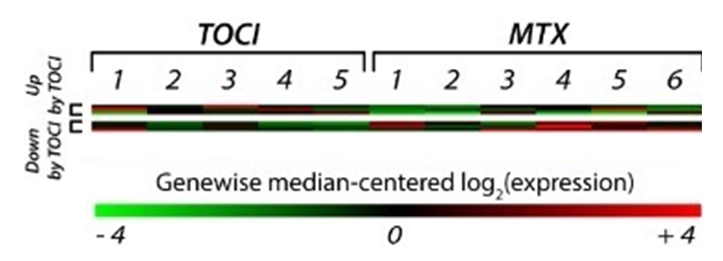

Supplement: S1 Fig — Each row represents the (log2) expression profile of a different DEG centred around its median value, while columns are individual patients. (TIF) [file pone.0282564.s005.tif]
